# A case of 18 years disease-free survival after combined pancreatoduodenectomy and hemicolectomy for carcinosarcoma of the transverse colon

**DOI:** 10.1186/s40792-021-01159-x

**Published:** 2021-03-25

**Authors:** Susumu Ohwada, Amika Moro, Nair Amit, Kazunari Sasaki, Shinji Sakurai, Atsuko Takada-Owada, Masaru Izumi, Yuhei Nakano, Yasushige Kashima, Hideki Matsuyama

**Affiliations:** 1 Center of Gastroenterology and Oncology, IMS Ota Chuo General Hospital, 875-1 Higashi-imaizumicho, Ota, 373-8513 Japan; 2 Department of General Surgery, Cleveland Clinic Foundation, Cleveland, OH USA; 3 Department of Pathology, Gunma CHUO General Hospital, Gunma, Japan; 4 Department of Pathology, Dokkyo Medical University Hospital, Tochigi, Japan; 5 Department of Surgery, Sudo Hospital, Gunma, Japan

**Keywords:** Carcinosarcoma, Region of pancreas head, Pancreatoduodenectomy with hemicolectomy

## Abstract

**Background:**

Ascertaining the origin of large tumors located in the region of the pancreas head and adjacent mesocolon can pose a challenge preoperatively. En bloc pancreatoduodenectomy with hemicolectomy is often required towards curative tumor resection (R0) of malignant tumors in this region.

**Case presentation:**

Herein we report a case of a 48-year-old man with two contiguous masses each 5 cm in size, located in the pancreatic head. The masses were detected incidentally by abdominal ultrasonography at an annual health check. Endoscopic biopsies revealed inflammation with no malignancy. Cross-sectional imaging showed the tumor direct invasion of the uncinate process of the pancreas, and the third portion of the duodenum. Based on imaging, a malignant submucosal tumor originating from mesenchymal cells in the mesentery of the transverse colon was made preoperatively. The mass required en bloc pancreatoduodenectomy, right hemicolectomy, and resection of the superior mesenteric vein. The final pathology was carcinosarcoma of the transverse colon. The patient survived 18 years after surgery without recurrence.

**Conclusions:**

Malignant tumors located in the region of the pancreas head should be considered for an en bloc curative tumor resection and adjuvant chemotherapy treatments offered that might be beneficial for carcinosarcoma.

## Background

Pancreatoduodenectomy (PD) is the mainstay of treatment for malignant tumors of the pancreatic head, lower common bile duct, and ampulla of the duodenum. Furthermore, locally advanced right-sided colon cancer (LARCC) may directly invade the duodenum or pancreatic head and requires combined PD with right hemicolectomy for achieving curative resection with clear (R0) margins [1–4]. In these instances, surgeons should make a deliberate operative plan to ensure negative resection margin while minimizing postoperative morbidity.

Carcinosarcomas of the colon and rectum are very aggressive tumors associated with poor prognosis, especially in tumors invading adjacent organs such as the duodenum, pancreas, and kidney [5]. We report our experience with such a case managed by aggressive surgical resection, with long-term survival and no local recurrence.

## Case presentation

A 48-year-old man without a significant past medical history was found to have an incidental upper abdominal tumor by abdominal ultrasonography at an annual health check. All laboratory examinations, including hemoglobin, albumin, pancreas enzyme, were within normal ranges. The carcinoembryonic antigen (CEA) concentration was slightly elevated as 4.6 ng/mL (< 2.5) without any smoking history. The carbohydrate antigen (CA19-9) concentration was within the normal range as 18 U/mL (< 37), as was DUPAN-2 (< 150 U/mL).

**Fig. 1:**
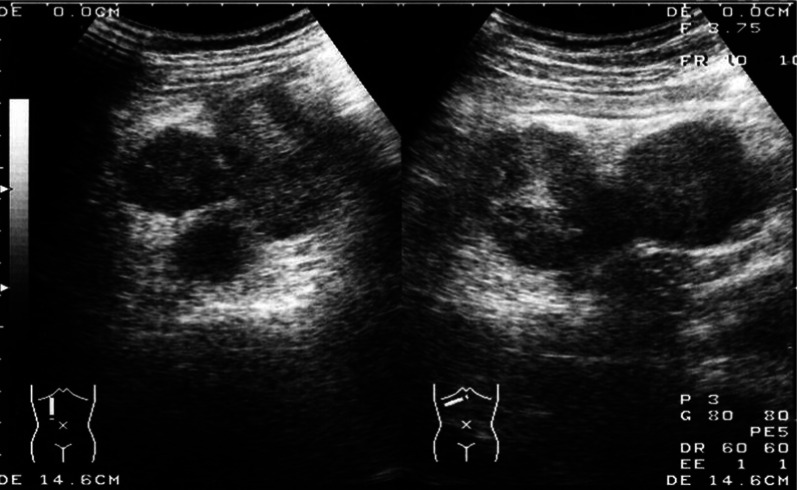
Ultrasonography showing two heterogeneous echogenic round contiguous masses, 5 cm in size each, at the region of the pancreatic head

**Fig. 2:**
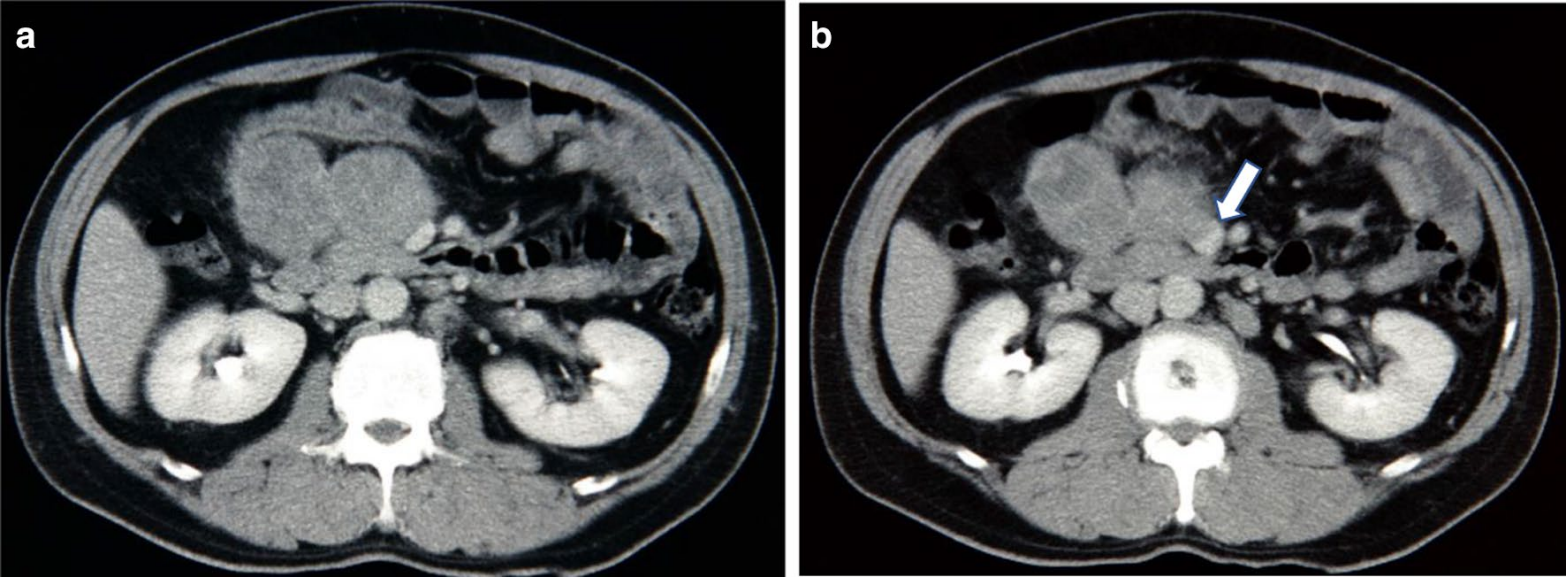
Abdominal computerized tomography (CT) showing two in-homogeneously enhanced round contiguous masses, 5 cm in size each, at the region of the pancreatic head (**a**), and the masses invading into the superior mesenteric vein (arrow) (**b**)

**Fig. 3:**
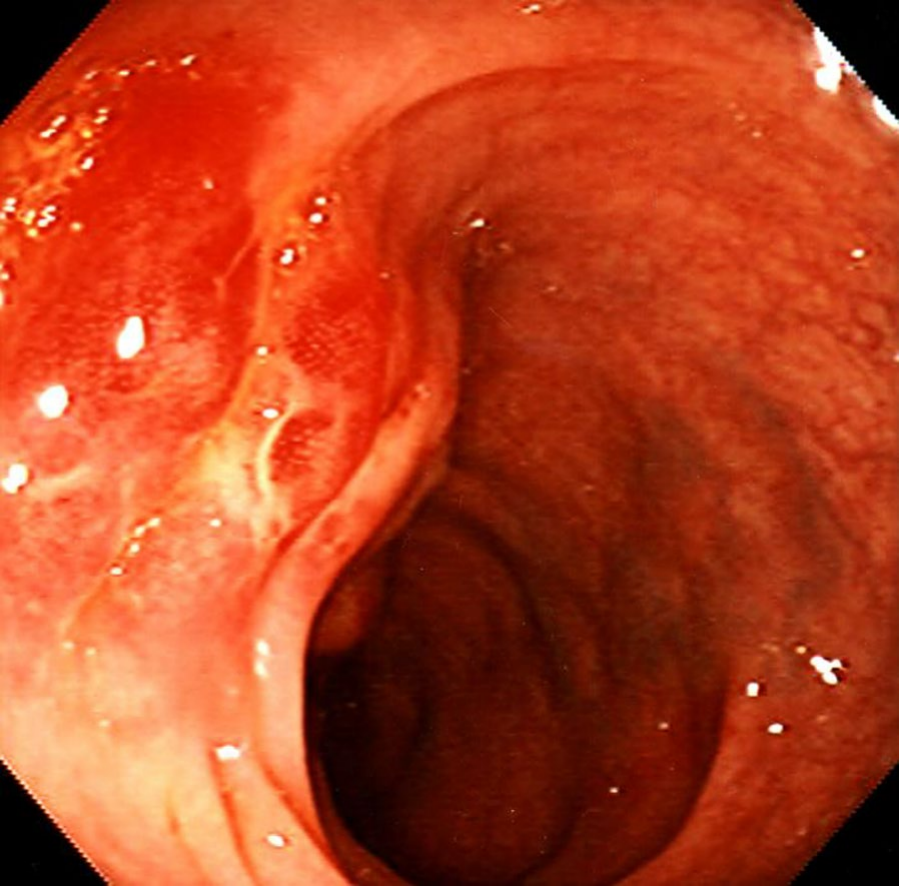
Colonoscopy demonstrating a red, raised wheal with a minute ulcer in the transverse colon, indicating submucosal invasion of an extramural tumor. Endoscopic biopsies revealed inflammation with no malignancy

**Fig. 4:**
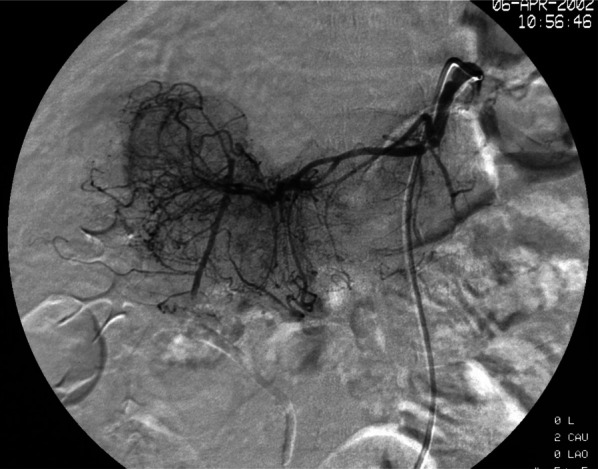
Angiograms showing a hyper-vascular tumor fed by the right branch of the middle colic artery

Ultrasonography showed two heterogeneous echogenic round contiguous masses, 5 cm in size each, at the region of the pancreatic head (Fig 1). Abdominal computerized tomography (CT) confirmed these findings (Fig 2a), which were in-homogeneously enhanced, and invading into the superior mesenteric vein (SMV) (Fig 2b). Colonoscopy demonstrated a red, raised wheal with a minute ulcer in the transverse colon, indicating submucosal invasion of an extramural tumor (Fig 3). Endoscopic biopsies revealed inflammation with no malignancy. Angiograms showed a hyper-vascular tumor fed by the right branch of the middle colic artery (Fig 4). The SMV was poorly visualized and narrowed. The tumor was not fed from any pancreatic or duodenal arteries. Upper gastrointestinal endoscopy showed no abnormality, including the papilla of Vater. Magnetic resonance cholangiopancreaticogram (MRCP) showed no abnormality in the pancreaticobiliary duct.

The mass was diagnosed as a malignant submucosal tumor originating from mesenchymal cells in the mesentery of the transverse colon, which directly invaded the duodenum or pancreatic head based on CT and colonoscopy findings although endoscopic biopsies could not get the malignancy. The patient underwent exploration towards curative-intent surgery and found to have a tumor mainly located in the mesentery of the transverse colon, which invaded both the uncinate process of the pancreas and the transverse colon. To achieve en bloc R0 resection, a combined PD, right hemicolectomy, and segmental cylindrical resection of the superior mesenteric vein (SMV) with lymph node dissection were performed and reconstruction was done with Billroth-I (Imanaga) reconstructive technique involving the gastric remnant, pancreatic duct, and biliary tree in that order.

**Fig. 5:**
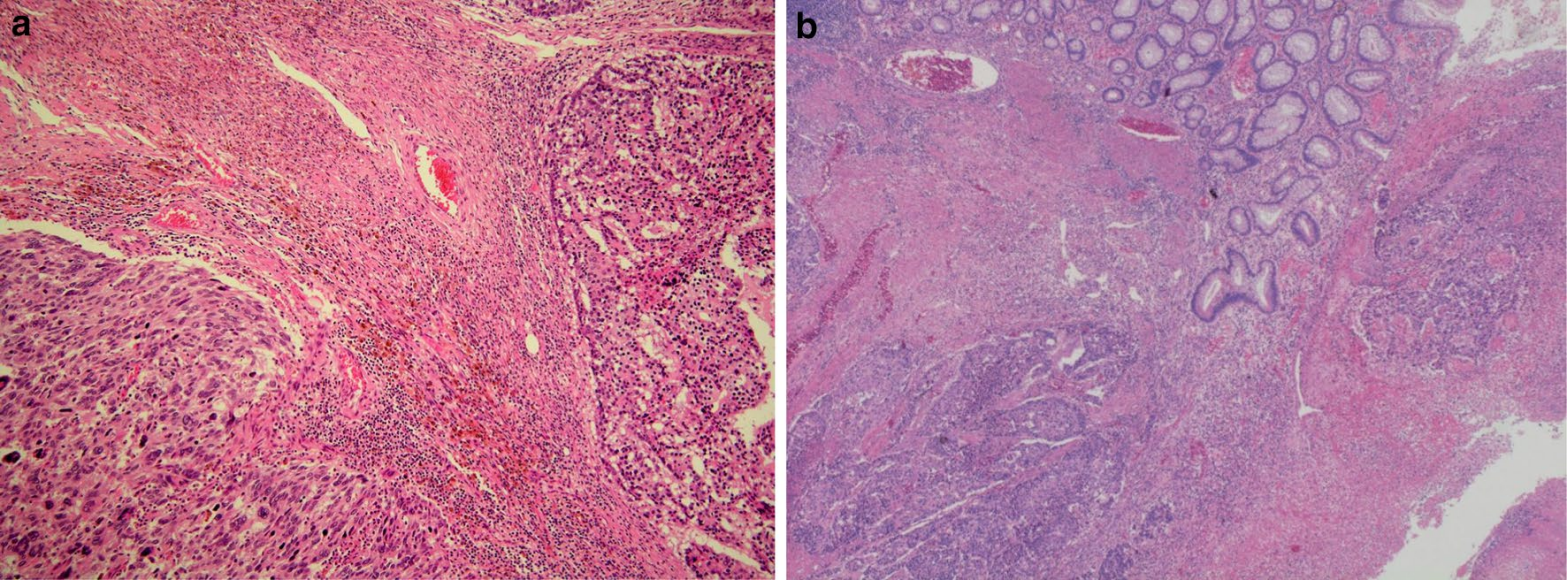
Histological examination revealing that a large part of the tumor was poorly differentiated adenocarcinoma, and pleomorphic sarcomatous tumor cells were seen in the part of hemorrhagic necrosis, where the transition of carcinomatous to sarcomatous tumor cells was observed (**a**). The carcinoma component was exposed in the ulcer base of the colon (**b**)

**Fig. 6:**
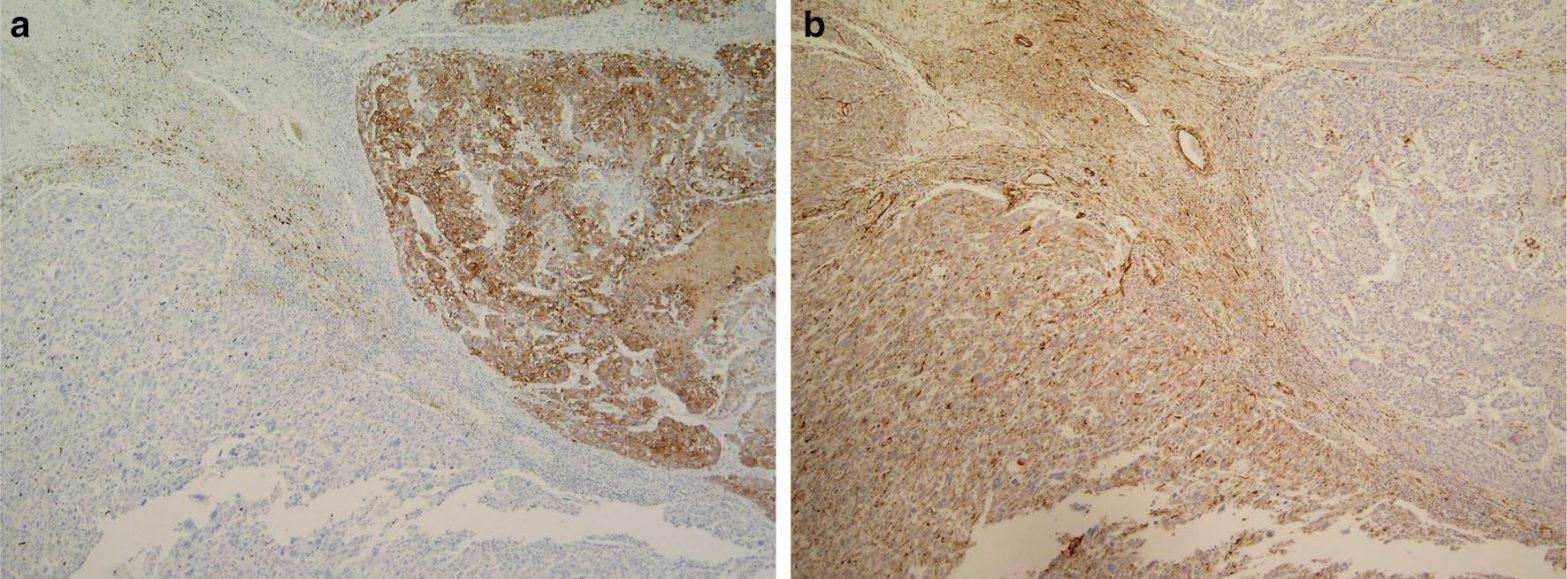
Immunohistochemically, the carcinomatous component was positive for CK7 (**a**), CK20, EMA, CDX2, AFP, and partly positive for vimentin. On the other hand, the spindle-shaped pleomorphic sarcomatous component was strongly positive for vimentin (**b**)

The resected tumor measured 9 × 7 × 6 cm. The cut section of the specimen showed a yellowish-white color with central necrosis. The tumor was mainly located in the wall of the transverse colon and colonic mesentery. Histological examination revealed that a large part of the tumor was poorly differentiated adenocarcinoma, and pleomorphic sarcomatous tumor cells were seen in the part of hemorrhagic necrosis, where the transition of carcinomatous to sarcomatous tumor cells was observed (Fig. 5a). The carcinoma component was exposed in the ulcer base of the colon (Fig. 5b). Immunohistochemically, the carcinomatous component was positive for CK7 (Fig. 6a), CK20, EMA, CDX2, AFP, and partly positive for vimentin. On the other hand, the spindle-shaped pleomorphic sarcomatous component was strongly positive for vimentin (Fig. 6b) and negative for CK7, CK20, EMA, CDX2, AFP, KIT, CD34, desmin, and S-100 protein. Based on, the histologic findings, and the immunohistochemistry results, the tumor was diagnosed as carcinosarcoma of the colon. Invasion of carcinosarcoma to the pancreas, duodenum, and SMV was not identified and just fibrous adhesion. Furthermore, there was no lymph node metastasis.

The postoperative course was uneventful. The patient received adjuvant chemotherapy with six cycles of 5-fluorouracil/leucovorin therapy based on the chemotherapy regimen for colon cancer. The patient has survived 18 years to the date after surgery with no recurrence.

## Discussion

This report describes a case of carcinosarcoma of the transverse colon, which was successfully treated by en bloc pancreatoduodenectomy and hemicolectomy. The endorsement points that the tumor originated from the transverse colon were as follows: (1) in the angiography, the tumor was fed by the right branch of the middle colic artery; (2) macroscopically, the tumor was mainly located in the wall of the transverse colon and colonic mesentery. Microscopically, the adenocarcinoma component exposed to the colonic lumen as a minimal ulcer, and invasion of carcinosarcoma to the pancreas and duodenum was not identified; (3) immunohistochemically, CDX2, a relatively specific marker of colorectal cancer, expressed in cancer cells; (4) the histological transition from adenocarcinoma to sarcoma component existed microscopically and immunohistochemically, denying the possibility of collision tumor of adenocarcinoma and sarcoma; (5) 18 years of survival with disease-free denies the possibility of metastatic tumor of other unknown tumor origins.

The imaging studies indicated that the tumor has a submucosal origin and malignant. Subsequently, GIST (gastrointestinal stromal tumor), leiomyosarcoma, malignant lymphoma, or Castleman’s lymphoma was listed in the differential diagnosis because of its size and specific properties. Firstly, mesocolic lymph nodal deposits from colon cancer were ruled out as colonoscopy did not identify colon cancer, and biopsies showed no malignancy. Also, these tumors were too large for lymph node metastasis, and additionally, there was no lymphadenopathy along the middle colic artery. Malignant lymphoma was deemed unlikely since the tumor was not located at a normal lymph node site along the main artery. GIST and leiomyosarcoma were ruled out because the growth patterns were different from this tumor. Although multiple imaging studies were conducted, the preoperative diagnosis of the present case was not definitive. Nonetheless, to obtain a definite diagnosis, endoscopic ultrasound-guided fine-needle aspiration (EUS-FNA)/biopsy (FNB) is an accurate and safe diagnostic modality, which is the first-line sampling procedure for histological/cytological diagnosis of solid pancreatic cancer [6]. Diagnostic laparoscopy can also be performed prior to surgical exploration for pancreatic mass, which could be another option [7].

For locally advanced tumors of the pancreas head, surgeons should make a deliberate operative plan to do an en bloc curative tumor resection (R0) and reduce postoperative morbidity. Local tumor growth into adjacent structures or organs does not necessarily preclude a curative resection, yet it may not be a standard procedure. Moreover, since PD and hemicolectomy are often culprits of significant morbidity that pose a surgical challenge, adequate surgical experience in tackling these scenarios is important.

In LARCC, the rate of malignant infiltration in adhesion between the tumor and adjacent organs has been reported to range between 71 and 94%. Furthermore, it is known that the preoperative CT and surgical exploration often cannot distinguish inflammatory adhesions from malignant infiltration of LARCC [4, 8]. In addition, the separation of colon cancer from the adherent organs may lead to tumor recurrence rates of 90–100% [9]. Therefore, to resect the malignant tumor in en bloc R0, our operative plan was PD combined with the resection of superior mesenteric vein and hemicolectomy. SMV resection for LARCC however, is still debatable. Locally advanced pancreatic cancer with less than 180°SMV contact [10] and without focal vessel narrowing is considered resectable. We conducted a segmental cylindrical resection of the SMV and reconstructed without a graft [11]. We believe that SMV resection under careful preparation to achieve R0 resection is achievable in such circumstances. In a previous study, a SMV resection for LARCC enabling a R0 resection, with no lymph node metastasis, was reported to have a favorable 5 years survival [12]. Finally, in this case, neither invasion of the carcinosarcoma to the pancreas, duodenum, and SMV was identified, nor lymph node metastasis was documented. Retrospective studies of LARCC show that histologic infiltration is seen in only 55–70% of cases in which the tumor is found to be adherent to the adjacent organs, whereas the rest represents a tumor fixation with inflammatory adhesions [13, 14]. Whether to attempt surgical dissection and separation of seemingly contiguously involved organs or not is a key consideration towards curative resection for cancer. If such adhesions between organs are due to cancer invasion, cancer cell seeding would occur in the surgical field or to the peritoneum. Therefore, surgeons should aim for en bloc resection as a default option for malignancy. When the tumor in the region of the pancreas head has been suspected of having infiltrated into adjacent organs, en bloc PD and hemicolectomy should be performed as long as this radical option is possible.

Previous reports indicated that most of the large bowel carcinosarcomas were sarcomatoid, poorly differentiated carcinoma [15]. In this case, the poorly differentiated carcinomatous component was positive for epithelial and colon cancer markers (CK7, CK20, EMA, CDX2, and AFP) and the spindle-shaped pleomorphic sarcomatous component was strongly positive for vimentin, but negative for epithelial markers. Thus, it was necessary to differentiate carcinosarcoma from collision tumor of carcinoma and sarcoma such as leiomyosarcoma and high-grade GIST. However, the sarcomatous component, in this case, was negative for other specific mesenchymal markers (KIT, CD34, desmin, and S-100 protein). In addition, the frequent histological transition from carcinomatous to sarcomatous tumor cells made a final diagnosis of carcinosarcoma developed from the transverse colon.

Both carcinosarcoma and sarcomatoid poorly differentiated carcinoma are aggressive tumors and have a poor prognosis [5]. Treatment for carcinosarcoma is by radical surgery and adjuvant chemotherapy, which should follow the guidelines for common colon adenocarcinomas. Adjuvant chemotherapy with six cycles of 5-fluorouracil/leucovorin therapy (in light of high-risk stage II diagnosis) was administered to this patient. A systematic review of PD and hemicolectomy for LARCC showed survival after resection was 55.2–66.3% at 5 years [2, 3]. Overall survival was greater in patients with no lymph node involvement than node-positive patients [2], and lymph node metastasis was the only independent predictor of poor survival on multivariable analysis [3]. Despite the diagnosis of carcinosarcoma of the transverse colon, our patient has survived 18 years because of a curative R0 en bloc PD, hemicolectomy and SMV resection, in addition to having no lymph node metastasis, and treatment with adjuvant chemotherapy. Therefore, an attempt should be made to perform a PD with SMV resection and hemicolectomy to achieve an R0 resection and reduce postoperative morbidities.

Carcinosarcoma of the colorectal has been reported in 25 cases in English literature including ours. The prognosis was so dismal that 14 among the 25 cases died within a 12-month period [5, 16]. Only one case survived longer than 5 years [17]. To our knowledge, disease-free survival of 18 years is the longest reported for a case of carcinosarcoma of the colorectal. Although chemotherapeutic agents for colorectal cancer have been given to carcinosarcoma as well, their usefulness has not been fully proven yet [18]. In the era of advanced chemotherapy and biotherapy, precision medicine might be able to improve the prognosis for carcinosarcoma of the colon.

## Conclusion

Malignant tumors located in the region of the pancreas head should be considered for en bloc curative tumor resection. This case was a tumor preoperatively diagnosed as a malignant mesenchymal tumor, which was treated by combining PD, hemicolectomy, and SMV resection. The final diagnosis was carcinosarcoma of the transverse colon. The patient was able to obtain an 18-year disease-free survival, which to our knowledge, is the longest in the case of carcinosarcoma of the transverse colon that has been reported. We report this case to improve the surgical management and consider the potential of chemotherapy treatments of this rare disease.

## Data Availability

Data sharing is not applicable to this article as no datasets were generated or analyzed during the current study.

## Abbreviations

PD: Pancreatoduodenectomy
LARCC: Locally advanced right colon cancer
CK7/20: Cytokeratin
EMA: Epithelial membrane antigen
SMV: Superior mesenteric vein
